# Health Seeking Behaviour among Medical Students in a Teaching Hospital of Nepal: A Descriptive Cross-sectional Study

**DOI:** 10.31729/jnma.4741

**Published:** 2020-01-31

**Authors:** Mukta Singh Bhandari, Jagdish Chataut

**Affiliations:** 1 Department of Community Medicine, Kathmandu University School of Medical Sciences, Dhulikhel, Kavrepalanchok, Nepal

**Keywords:** *health care utilization*, *medical students*, *health behaviour*

## Abstract

**Introduction:**

Medical students are more prone to suffer from various physiological and psychological problems but rather than seeking for formal health care, they tend to do informal consultation and often practice self medication. Thus, this study aimed to find out the health seeking behavior of medical students.

**Methods:**

This descriptive cross-sectional study was done among first and second year medical students of a teaching hospital from September to November 2019 after taking ethical approval from Institutional Review Committe. Total of 235 students were included in the study and self administered questionnaire was used. Data entry and analysis was done using Statistical Package for Social Sciences version 20.0.

**Results:**

Among 235 students who participated in the study, 172 (73%) reported having health problems in the last 12 months, and fever and headache were commonly reported by 21 (13%) and 18 (50%) students, respectively. Total of 112 (65%) students visited hospital/clinic for health problems and reason given for not visiting hospital/clinic was 12 (28%) thinking that the problem was minor. University hospital was the most preferred place 189 (80%) during health problem and parents were the first people for consultation 116 (49%). Mean duration of absenteeism was 2.17±4.1 days and 167 (88%) visited hospital more than five times.

**Conclusions:**

Health problems were common among students and most of them required multiple hospital visits. Many students seeked for health from hospital/ clinic but informal consultations were also seen.

## INTRODUCTION

Health seeking behaviour is a response to illness as a result of which person undertakes activities in order to find remedy for the problem or prevent illness.^1,2^ Adolescents and young people are usually thought of as a healthy group but they tend to have various health problems like depression, stress, anxiety and reproductive health problems which are often hidden or under diagnosed.^3–5^ And young medical students are more prone to it but often found to do informal consultation^6–10^ and practice self diagnosis and/ or self medication.^11–13^

Health seeking behaviour not just affects the present but also determines the care seeking and care giving practice of medical students in the future. Studies about self medication practice among medical students have been done in Nepal^14,15^ but there is dearth of study about health seeking behaviour.

Thus, this study aims to find out the health seeking behaviour of medical students.

## METHODS

This was a descriptive cross-sectional study done at Kathmandu University School of Medical Sciences (KUSMS). Ethical approval was taken from Institutional Review Committee of Dhulikhel Hospital, KUSMS (Reference number 123/17). The aims and objectives of the study along with information about confidentiality and voluntary participation were explained to the students in group before questionnaire was distributed. Written consent was taken from each respondent. Those students who were absent during the day of data collection were followed up later. The duration of study was from September to November 2019.

All the students studying in first and second year of MBBS and BDS were eligible for the study which gave the sample size of 251.

Data was collected using questionnaire which was pretested among Bachelor of Physiotherapy students and needful correction were done. The questionnaire included information about socio-demographic profile, health problems and health care utilization during last episode of illness within 12 months and health seeking practice.

A descriptive analysis of socio-demographic variables was done using mean, frequency, percentage and standard deviation. Data entry and analysis was done in Statistical Package for Social Sciences version 20.

## RESULTS

A total of 235 students participated in the study. The mean age of student was 20.11 ±1.858 years (Mean±SD) and total 228 (97%) students followed Hindu religion. One hundred and forty students (60%) were from MBBS and 95 (40%) from BDS stream and 124 (53%) were female. Total 218 (93%) students were Nepalese nationals and 162 (69%) resided in hostel. Only 97 (41%) students said that there was health related personnel in their family (Table 1).

**Table 1:** Socio-demographic profile of respondents.

Socio-demographic variables		Frequency n (%)
Sex of respondents	Male	111 (47)
	Female	124 (53)
Religion	Hindu	228 (97)
	Buddhist	4 (2)
	Christian	3 (1)
Nationality	Nepalese	218 (93)
	Indian and others	17 (7)
Place of residence	Home	33 (14)
	Hostel	162 (69)
	Rent and others	40 (17)
Health related personnel in the family	Yes	97 (41)
	No	138 (59)

**Figure 1.**
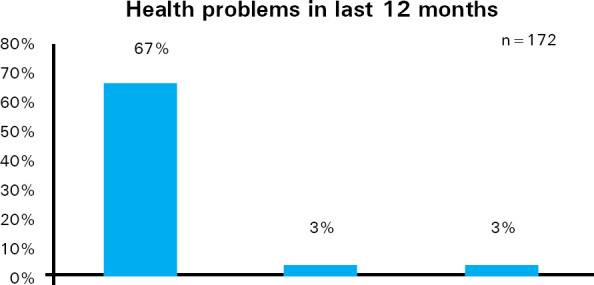
Health problems in last 12 months.

Out of 235 respondents, 172 (73%) said that they had health related problems in the last 12 months. Out of 172 respondents who reported having any problem, 158 (67%) said that the most common problem was physical problem (Figure 1).

The students were asked about the main symptoms that they had experienced in the past 12 months. The symptoms were classified into system during analysis which found that 79 (50%) students had respiratory symptoms, 30 (19%) students had gastrointestinal symptoms, 26 (16.5%) students had musculoskeletal symptoms and 23 (14.5%) students had symptoms related to other systems and miscellaneous. Miscellaneous symptoms included burning micturition, mass, vertigo and weight gain. Among all, headache and fever were most commonly reported by 21 (13%) and 18 (11.4%) respectively (Table 2).

**Table 2:** Main physical symptoms reported by respondents.

Main physical symptoms		n (%)
Respiratory	Headache	21 (13.3)
	Fever	18 (11.4)
	Cough	15 (9.5)
	Coryza	13 (8)
	Sore throat	12 (7.6)
Gastrointestinal	Abdominal pain	15 (9.5)
	Loose stool	7 (4.5)
	Nausea and vomiting	7 (4.5)
	Constipation	1(0.5)
Musculoskeletal	Body pain	15 (9.5)
	Sprain or injury	7 (4.5)
	Weakness and fatigue	4 ( 2.5)
Other systems	Skin	7 (4.5)
	Eye and vision	5 (3)
	Menstrual	4 (2.5)
	Dental	3 (2)
	Miscellaneous	4 (2.5)

**Figure 2.**
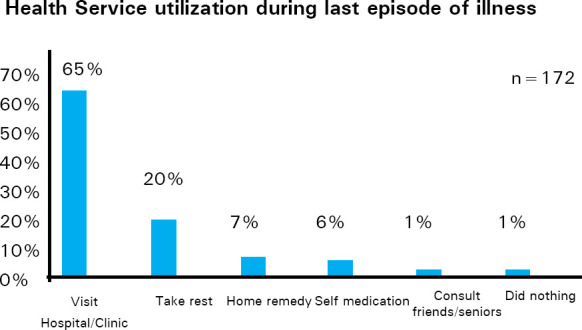
Health service utilization during last episode of illness.

Out of 172 respondents who had health problems, only 112 (65%) visited hospital/ clinic during last episode of illness while one (1%) reported consulting friends/ senior informally and one (1%) reported doing nothing (Figure 2).

**Figure 3.**
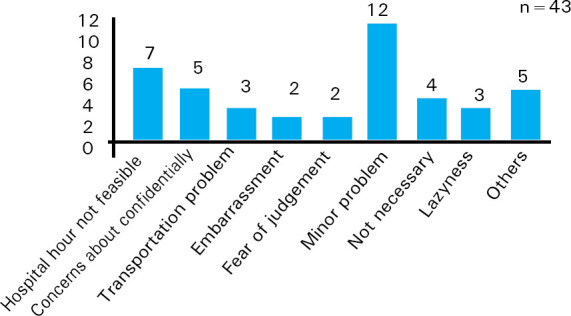
Reasons for not visiting hospital/clinic.

The most common reason given for not visiting hospital/ clinic was respondents thinking that the problem was minor 12 (28%) while five (12%) respondents gave other reasons which included home remedy more effective 2 (5%), self limiting problem 2 (5%) and took previously prescribed medication 1 (2%) (Figure 3).

Total 189 (80%) students said that university hospital was the first place that they would seek for help during health problems followed by other hospitals 25 (11%), clinic 12 (5%) and pharmacy 9 (4%). One hundred and sixteen (49%) students said that they consulted their parents during episode of illness while 69 (29%) consulted their friends, 46 (20%) consulted doctors directly while 2 (1%) consulted their seniors informally. Only two respondents (1%) said that they would consult other persons which included husband and no one.

When asked about absenteeism due to any health problems, 51% reported to have been absent in the past 12 months and the mean duration of absenteeism was 2.17±4.1 days (Mean±SD). Though, only 172 (73%) students reported having any problem in the past 12 months, 190 (81%) of them said that they had visited hospital due to any health related problems. Out of 190 students, 167 (88%) said that they had visited hospital less than five times while 23 (12%) reported to have visited hospital more than five times in the previous year.

## DISCUSSION

The results of this study indicate that medical students commonly suffer from physical health problems which are consistent from previous studies done among medical students in Goa^12^ and Nigeria^11^ in which 80.4% and 98.7% of the students reported some form of illness. But psychological problem was very less reported in our study compared to studies done by Adhikary et al. in KIST, Nepal^16^ in which 29% of the students had depression, 24% had somatic problems^16^ and Venkatarao et al. in Bhubaneswor, India^17^, in which more than half of the students suffered from anxiety (66.9%), depression (51.3%) and stress (53%). The poor reporting of mental health problems in our study may be due to fear of confidentiality and judgement as the physicians were teachers too.

In our study, most of the students visited hospital/ clinic during last episode of illness and very few did self medication or used home remedy which is in contrast from the studies done among medical students of Lumbini,^14^ Goa,^12^ Belgrade,^18^ Sharjah^19^ and Nigeria^11^ in which very high rates of self medication were found. Though, consulting friends or seniors is common among medical students, it was less common in our study group which may be because our study group consisted of first and second year students only and they were located far from senior student's block and might have less contact.

The most common barrier for not seeking formal health care was thinking that their problem was minor which was consistent with the finding from study done by Sawalha K et al in UAE, in which most of the students tend to think that their problem was minor and ignored it.^20^ Similar findings were seen in study done by Ajaegbu OO and Ubochi II in Nigeria in which students preferred not to do anything as they perceived it as not life threatening and also due to reluctance.^11^

Our study found out that absenteeism was common as in studies from Goa,^12^ Pakistan^20^ and Ethiopia^21^ but it was lower compared to their findings.

This study included only MBBS and BDS first and second year students of KUSMS, thus the findings may not represent behaviour and practice of all medical students. Information bias is also expected in the study due to fear of judgement.

## CONCLUSIONS

Health seeking behaviour of medical students was favourable with most of the students visiting hospital/clinic but the first person to consult was commonly parents rather than health personnel. Perceiving health problems as minor and no feasible time for check up for students were common barriers for health care seeking. Thus, by providing favourable environment and feasible hospital hour, health care seeking of medical students can be improved.
